# Distinguishing Kikuchi-Fujimoto disease from lymphoma in patients by clinical and PET/CT features

**DOI:** 10.1097/MD.0000000000037779

**Published:** 2024-04-19

**Authors:** Mu-Hua Cheng, Liang-Jun Xie

**Affiliations:** a Department of Nuclear Medicine, The Third Affiliated Hospital of Sun Yat-Sen University, Guangzhou, Guangdong, China.

**Keywords:** 18F-fluorodeoxyglucose, Kikuchi-Fujimoto disease, lymphadenopathy, lymphoma, positron emission tomography/computed tomography

## Abstract

To develop a scheme for distinguishing Kikuchi-Fujimoto disease (KFD) from lymphoma in patients presenting enlarged lymph nodes (LNs) predominantly on the upper side of the diaphragm. From November 2015 to August 2023, 32 KFD patients and 38 lymphoma patients were pathologically confirmed and enrolled in this retrospectively study. Clinical and ^18^F-fluorodeoxyglucose positron emission tomography (PET)/computed tomography (CT) features were collected. When comparing those PET/CT parameters, we set 5 models with different research objects: (1) all affected LNs; (2) the 5 largest affected LNs in terms of maximum diameter; (3) the 5 largest affected LNs in terms of maximum standard uptake values (SUVmax); (4) the largest affected LNs in terms of maximum diameter; (5) the largest affected LNs in terms of SUVmax. Compared to lymphoma patients, KFD patients were younger; and with higher incidence of fever, arthralgia, abnormal serum white blood cell, lactate dehydrogenase (LDH) and splenomegaly; lower incidence of affected LNs perinodal infiltration, necrosis and conglomeration; more affected LNs in *Head and Neck* nodes (particularly in level II) and *Axillary* in KFD (*P* ˂ .05). PET/CT parameters presented as various difference in each model. Finally, 11 clinical and PET/CT features (age ≤ 34, with fever, arthralgia, abnormal white blood cell, abnormal LDH, and without node necrosis and node conglomeration have a score of 2 each; splenomegaly, perinodal infiltration, median maximum diameter ≤ 20.5 and median SUVmax ≤ 7.1 of affected LNs in model 2 have score of 1 each) were selected as scheme items for distinguishing KFD from lymphoma. Individuals who have a total score > 8, meet the criteria for KFD. Sensitivity and specificity were high: 86.8% (95% CI: 71.9%, 95.5%) and 96.9% (95% CI: 83.7%, 99.5%), AUC = 0.975 (95% CI: 90.5%, 99.6%), respectively. It can effectively distinguish KFD from lymphoma by clinical and PET/CT parameters.

## 1. Introduction

Kikuchi-Fujimoto disease (KFD), also known as histiocytic necrotizing lymphadenitis, is a rare, benign, self-limiting condition characterized by subacute necrotizing regional lymphadenopathy with tenderness, predominantly in cervical region.^[1]^ In spite of many studies in the literature, the etiology and pathogenesis of KFD remain unclear. Clinicians are unfamiliar with this entity, which can pose significant diagnostic challenges as it can easily be confused with other lymph nodes (LNs) enlargement diseases, such as lymphomas.^[1]^

Many Clinicians have difficulty accurately diagnosing KFD based on physical findings, laboratory findings or imaging alone. Misdiagnosis of KFD is reported to occur in up to 40% of patients.^[2]^ KFD commonly affects young adults and is frequently associated with mild fever, and occasionally with other systemic symptoms such as weight loss, headache, arthralgia, and upper respiratory symptoms.^[1]^ Hepatomegaly and splenomegaly rarely occur. Diagnostic laboratory findings include mild anemia, elevated C-reactive protein, leukopenia and leukocytosis, elevated serum lactate dehydrogenase (LDH).^[1]^ It was reported that more than 5 nodal levels or bilateral neck LNs can be affected in KFD patients. Moreover, the affected LNs were with no or minimal nodal necrosis, marked perinodal infiltration.^[3]^ Other article reported that KFD were with multiple enlarged LNs usually in unilateral cervical distributing at levels II–V of the cervical neck.^[4]^ Although various contrast-enhanced computed tomography (CT) findings for LNs in the neck have been reported,^[4–6]^ no any definitive imaging modality for distinguishing KFD and malignancy has been identified. Due to its nonspecific clinical, laboratory and imaging manifestations, KFD can easily be confused with lymphoma, especially in which lesion predominantly in cervical region and the early lesion lacking overt necrosis.

^18^F-fluorodeoxyglucose (^18^F-FDG) positron emission tomography (PET)/CT is a widely used imaging modality in oncology based on the elevated glucose metabolism that is present in tumor cells. Recently, a few case reports and small studies of ^18^F-FDG PET/CT findings in patients with KFD have been reported.^[5,7–10]^ These reports have suggested that patients with KFD exhibit a high FDG uptake in their affected LNs. However, the significances of ^18^F-FDG PET/CT imaging findings have yet to be determined. Therefore, the purpose of this study was to determine the optimal clinical and PET/CT features for differentiating KFD and lymphoma on the condition that patients presenting enlarged LNs predominantly on the upper side of the diaphragm.

## 2. Methods

### 2.1. Patients

A search of our institution’s pathology, radiology, and medical records between November 2015 to August 2023 revealed 32 KFD patients and 38 lymphoma patients (consisting of 2 T-cell, 23 B-cell non-Hodgkin lymphoma and 13 Hodgkin lymphoma patients) whose data were considered for inclusion in this retrospective study. Consent forms were obtained from all patients. Those patients underwent ^18^F-FDG PET/CT and presented enlarged LNs predominantly on the upper side of the diaphragm, and had pathologically confirmed KFD or lymphoma in the same admission period. Exclusion criteria were as follows: lack of pathologic diagnosis, main affected region other than upper side of the diaphragm, with other LNs enlargement diseases, or lymphoma with solid organ involvement. Clinical characteristics, associated symptoms, and laboratory study results were collected from the medical records (Table 1). The study followed the 1964 Helsinki Declaration and its later amendments or comparable ethical standards.

**Table 1:** Comparison of the patient characteristics in Kikuchi-Fujimoto disease (KFD) and lymphoma.

Characteristics	KFD (n = 32)		Lymphoma (n = 38)		*χ* ^2^	*P* *	r	*P* †
Sex (female/male)	25/7		26/12		0.827	.363		
Age [year, median (25th-75th percentiles)]	27.0 (21.0–33.5)		43.5 (35.8–63.3)		–4.647	<.001	0.560	<.001
*Systemic symptoms* (–/+, %)								
Fever	3 (9.4)	29 (90.6)	29 (81.6)	7 (18.4)	36.255	<.001	–0.720	<.001
Arthralgia	23 (71.9)	9 (28.1)	38 (100)	0 (0)	12.264	<.001	–0.419	<.001
Lymph node pain	28 (87.5)	4 (12.5)	33 (86.8)	5 (13.2)	0.007	.935		
Skin temperature arisen	31 (96.9)	1 (3.1)	35 (92.1)	3 (7.9)	734	.392		
*Laboratory findings* (–/+, %)								
Abnormal WBC	9 (28.1)	23 (71.9)	29 (76.3)	9 (23.7)	16.483	<.001	–0.485	<.001
Abnormal RBC	23 (71.9)	9 (28.1)	25 (65.8)	13 (34.2)	0.299	.585		
Abnormal platelet	24 (75.0)	8 (25.0)	31 (81.6)	7 (18.4)	0.447	.504		
Abnormal LDH	5 (15.6)	27 (84.4)	38 (73.7)	10 (23.7)	23.499	<.001	–0.579	<.001
*PET/CT parameters* (–/+, %)								
Hepatomegaly	21 (65.6)	11 (34.4)	39 (76.3)	9 (23.7)	0.973	.334		
Splenomegaly	6 (18.8)	26 (81.3)	16 (42.1)	22 (57.9)	4.397	.036	–0.251	.036
Perinodal infiltration	27 (84.4)	5 (15.6)	23 (60.5)	15 (39.5)	4.841	.028	0.263	.028
Node necrosis	31 (96.9)	1 (3.1)	24 (63.2)	14 (36.8)	11.729	.001	0.409	<.001
Node conglomeration	30 (93.8)	2 (6.3)	17 (44.7)	21 (55.3)	18.916	<.001	0.520	<.001

LDH = lactate dehydrogenase, PET/CT = positron emission tomography/computed tomography; categorical variables are presented as numbers (percentage), RBC = red blood cell, WBC = white blood cell.

* Pearson Chi-Square test.

† Bivariate correlation analysis.

### 2.2. PET/CT technique and evaluation

For PET/CT scanning, each patient had fasted for more than 4 hours, and had a pre-scan blood glucose analysis to ensure a level < 200 mg/dL. Subsequently, a dose of 370 MBq of ^18^F-FDG was administered. One hour after injection, FDG images were acquired from the vertex to the upper thighs using a GE Discovery PET/CT Elite system. CT scan (voltage: 120 kV, current: 100 mAs, 5 mm layer, 512 × 512 matrix, and 60 cm FOV) was firstly performed and then PET (9–10 beds, 3 minutes/bed). After reconstruction, image analysis was processed using GE postprocessing fusion software.

Two experienced nuclear medicine physicians interpreted the images. Affected LNs were included when one of the following was present: (1) increased size (maximum diameter compared to the normal size of the respective region), (2) abnormal shape (not oblong or lima bean-shaped but spherical), (3) clusters of small lymph nodes, (4) necrosis, (5) perinodal fat infiltration, (6) positive FDG uptake. Regions of interests were drawn to correspond with the affected LNs in transverse section CT image. The locations, FDG avidity and size factors (maximum diameter, orthogonal diameter, and axial ratio) of the affected LNs, as well as characteristics including the presence of necrosis, perinodal infiltration and conglomeration were systematically evaluated in either *Head and Neck, Axillary, Supraclavicular, Mediastinal* or *Chest Wall* (unilateral or bilateral). We recorded the locations of LNs according to previously reported.^[11–13]^ The average CT value (CTavg) and maximum standard uptake values (SUVmax) in the regions of interests were then recorded. In addition, the information about liver and spleen were evaluated.

The maximum diameter, orthogonal diameter, axial ratio, CTavg and SUVmax of each affected LNs were recorded. When comparing those PET/CT parameters between the 2 disease, we set 5 models with different research objects for comparison: (1) all affected LNs in each patient; (2) the 5 largest affected LNs in terms of maximum diameter in each patient; (3) the 5 largest affected LNs in terms of SUVmax in each patient; (4) the largest affected LNs in terms of maximum diameter in each patient; (5) the largest affected LNs in terms of SUVmax in each patient.

### 2.3. Testing and adaptation of the differential diagnostic scheme

Based on results from exploratory analyses, we defined a rough differential diagnostic scheme focusing on the items selected by association analysis. The scheme was defined based on scores computed as weighted sums of binary indicators of presence/absence of items. Cutoff values for case designation for rough scheme were computed using receiver operating characteristic (ROC) methods.

### 2.4. Statistics

All patients were divided into 2 groups consisting of KFD and lymphoma. The parameters of clinical features and PET/CT imaging features were compared between the 2 groups by one-way analysis of variance with a post hoc Games-Howell test, which has robustness for analysis even in unequal variance. The Mann–Whitney *U* test was performed to analyze differences between the 2 groups, while the χ^2^ test or Fisher exact test was performed on categorical data. Bivariate correlation analysis and one-way analysis of variance test were performed to analyze connections between disease type and clinical characteristics and PET/CT parameters. Differences were considered to be statistically significant at a 2-sided *P* value of <0.05. Statistical analysis was performed using a software package (SPSS software, version 16.0; SPSS Inc., Chicago, IL). ROC curves were plotted using MedCalc statistical software (Version 9.2.1.0).

## 3. Results

### 3.1. Patient characteristics

The characteristics of the 32 patients with KFD and 38 patients with lymphoma are summarized in Table 1. It showed that patients with KFD seem to be younger than patients with lymphoma (27.0 vs 43.5, *P* < .001). 90.6% patients with KFD in our study presented with a fever (temperature > 37.3 °C, 39.3 ± 0.7 °C), however, the proportion was smaller in patient with lymphoma (18.4%) (*P* < .001). The incidence of arthralgia (28.1% vs 0%, *P* < .001), abnormal serum white blood cell (WBC) (75.0% vs 26.3%, *P* < .001), LDH (84.4% vs 23.7%, *P* < .001) in KFD was higher than lymphoma. In KFD patients, 15 of 32 (46.9%) with leukopenia (2.3 × 10^9^/L ± 0.7), and 8 of 32 (25.0%) with leukocytosis (14.5 × 10^9^/L ± 3.4). In the lymphoma patients, 3 of 38 (7.9%) with leukopenia (2.2 × 10^9^/L ± 0.5), and 6 of 38 (15.8%) with leukocytosis (19.6 × 10^9^/L ± 22.3). The incidence of abnormal red blood cell (RBC), abnormal platelet, lymph node pain or skin temperature arisen between the 2 type patients were with no significant differences (*P* > .05).

### 3.2. Comparison of PET/CT parameters according to the disease type (KFD or lymphoma)

The incidence of splenomegaly in KFD was higher than in lymphoma patients (81.3% vs 57.9%, *P* = .036) (in Table 1). Most patients with KFD or with lymphoma were without hepatomegaly, however, there were no significant differences of those result between the 2 type patients (*P* > .05). More lymphoma patients with affected LNs perinodal infiltration (39.5% vs 15.6%, *P* = .028), necrosis (36.8% vs 3.1%, *P* = .001) and conglomeration (55.3% vs. 6.3%, *P* < .001) than KFD patients.

The total number of affected LNs were 2240 and 1799 in patients with KFD and in patients with lymphoma, respectively. The location of affected LNs of patients with KFD or lymphoma are summarized in Table 2. The composition ratio of affected LNs in *Head and Neck, Axillary, Supraclavicular, Mediastinal* and *Chest Wall* were 75.0%, 25.0%, 12.5%, 8.3%, and 5.0% in patients with KFD and 61.9%, 30.4%, 1.9%, 5.4%, and 0.4% in patients with lymphoma, respectively. The affected LNs in patients with KFD were predominantly in *Head and Neck* region, in which level II was the most frequent (38.5%), followed by level V (21.5%). Furthermore, the number of affected LNs were larger in level of right II (5.0 vs 2.0, *P* = .001), left II (10.0 vs 1.5, *P* < .001), left III (2.5 vs 0.0, *P* = .025) and left V (3.5 vs 1.5, *P* = .025) from *Head and Neck* nodes and right I (6.0 vs 0.0, *P* = .002), left I (5.5 vs 0.0, *P* = .012) from *Axillary* in patients with KFD than in patients with lymphoma. However, there were no significant differences from *Supraclavicular, Mediastinal*, or *Chest Wall* affected LNs between the patients with KFD and the patients with lymphoma (*P* > .05).

**Table 2:** Comparison of the affected lymph nodes number in each location in Kikuchi-Fujimoto disease (KFD) and lymphoma [median (25th–75th percentiles)].

Node anatomy	KFD	Lymphoma	*P*		KFD	Lymphoma	*P*
*Head and Neck nodes*							
Right occipital	0.0 (0.0–0.5)	0.0 (0.0–0.0)	.069	Left occipital	0.0 (0.0–0.0)	0.0 (0.0–0.0)	.341
Right intraglandular parotid	0.0 (0.0–2.0)	0.0 (0.0–1.0)	.321	Left intraglandular parotid	0.0 (0.0–3.0)	0.0 (0.0–0.0)	.014
Right retropharyngeal	0.0 (0.0–0.0)	0.0 (0.0–0.0)	1.000	Left retropharyngeal	0.0 (0.0–0.0)	0.0 (0.0–0.0)	.556
Right I	1.0 (0.0–3.0)	0.0 (0.0–3.0)	.336	Left I	1.5 (0.0-3.0)	0.0 (0.0–2.0)	.040
Right II	5.0 (3.0–12.0)	2.0 (0.0–6.0)	.001	Left II	10.0 (3.0–14.0)	1.5 (0.0–3.0)	<.001
Right III	1.0 (0.0-3.0)	1.0 (0.0–3.0)	.796	Left III	2.5 (1.0-4.0)	0.0 (0.0–3.0)	.025
Right IV	1.0 (0.0–3.0)	1.5 (0.0–3.0)	.636	Left IV	2.0 (0.0-4.0)	1.5 (0.0–3.0)	.321
Right V	2.0 (0.0–4.5)	2.0 (0.0–4.0)	.842	Left V	3.5 (2.0-10.5)	1.5 (0.0–6.0)	.025
Right VI	0.0 (0.0–1.0)	0.0 (0.0–0.0)	.607	Left VI	0.0 (0.0–0.0)	0.0 (0.0–0.0)	0.757
*Axillary lymph nodes*							
Right I	6.0 (1.5–14.0)	0.0 (0.0–6.0)	.002	Left I	5.5 (1.5–14.5)	0.0 (0.0–7.0)	.012
Right II	0.0 (0.0–2.0)	0.0 (0.0–0.0)	.038	Left II	0.0 (0.0–2.0)	0.0 (0.0–1.0)	.365
Right III	0.0 (0.0–1.0)	0.0 (0.0–1.0)	.588	Left III	0.0 (0.0–0.0)	0.0 (0.0–0.0)	.476
*Supraclavicular nodes*							
Right supraclavicular	0.0 (0.0–0.0)	0.0 (0.0–0.0)	.729	Left supraclavicular	0.0 (0.0–1.0)	0.0 (0.0–1.0)	.989
*Mediastinal nodes*							
Right paratracheal	1.0 (0.0–3.0)	0.0 (0.0–4.0)	.850	Left paratracheal	0.0 (0.0–0.0)	0.0 (0.0–0.0)	.663
Right hilar	0.0 (0.0–1.0)	0.0 (0.0–0.0)	.358	Left hilar	0.0 (0.0–1.0)	0.0 (0.0–0.0)	.087
Prevascular	0.0 (0.0–0.0)	0.0 (0.0–3.0)	.056	Subcarinal	0.0 (0.0–1.5)	0.0 (0.0–0.0)	.004
Para-aortic	0.0 (0.0–0.0)	0.0 (0.1–0.9)	.537	Subaortic	0.0 (0.0–0.0)	0.0 (0.0–0.0)	.398
Right pulmonary ligament	0.0 (0.0–0.0)	0.0 (0.0–0.0)	.860	Left pulmonary ligament	0.0 (0.0–0.0)	0.0 (0.0–0.0)	.902
*Chest wall nodes*							
Right internal mammary	0.0 (0.0–0.0)	0.0 (0.0–0.0)	.191	Left internal mammary	0.0 (0.0–0.0)	0.0 (0.0–0.0)	.061
Right cardiophrenic	0.0 (0.0–0.0)	0.0 (0.0–0.0)	.497	Left cardiophrenic	0.0 (0.0–0.0)	0.0 (0.0–0.0)	.061
Right retrocrural	0.0 (0.0–0.0)	0.0 (0.0–0.0)	.359	Left retrocrural	0.0 (0.0–0.0)	0.0 (0.0–0.0)	1.000

The Mann–Whitney *U* test was performed to analyze differences between the 2 groups.

The maximum diameter, orthogonal diameter, axial ratio, CTavg and SUVmax of each affected LNs were recorded. The value ranged from 2 mm to 27 mm, 1 mm to 17 mm, 0.2 to 1.0, –90 HU to 115 HU, 0.4 to 50.0 in patients with KFD and from 3 mm to 124 mm, 2 mm to 100 mm, 0.3 to 1.0, –70 HU to 98 HU, 0.1 to 63.0 in patients with lymphoma, respectively. Compared to patients with lymphoma, in model 1 and model 2, the maximum diameter, orthogonal diameter, axial ratio, CTavg, and SUVmax were all smaller or lower in patients with KFD (*P* < .05) (Table 3). Furthermore, maximum diameter and orthogonal diameter were intensely related to the disease type in model 2. In model 3, the maximum diameter, orthogonal diameter, axial ratio were all smaller in patients with KFD (*P* < .05). In model 4, the maximum diameter, orthogonal diameter, axial ratio and SUVmax were all smaller or lower in patients with KFD (*P* < .05). In model 5, the maximum diameter, orthogonal diameter were all smaller in patients with KFD (*P* < .05).

**Table 3:** Comparison of the PET/CT parameters in Kikuchi-Fujimoto disease (KFD) and lymphoma [median (5th–25th–75th–95th percentiles)].

Parameters	KFD	Lymphoma	z	*P* *	F	*P* †	Eta squared
*Model* 1							
Maximum diameter (mm)	9.0 (4.0–7.0–11.0–16.0)	11.0 (5.0–8.0–15.7–27.0)	–18.606	<.001	365.648	<.001	0.083
Orthogonal diameter (mm)	6.0 (3.0–4.0–7.0–11.0)	8.0 (4.0–6.0–11.0–20.0)	–23.434	<.001	477.999	<.001	0.106
Axial ratio	0.7 (0.4–0.6–0.8–1.0)	0.7 (0.5–0.6–0.8–1.0)	–8.666	<.001	75.197	<.001	0.018
CTavg (HU)	40.0 (10.0–30.0–47.0–58.0)	44.0 (16.0–35.0–51.0–62.0)	–10.556	<.001	88.318	<.001	0.021
SUVmax	3.5 (1.1–2.2–5.6–11.3)	4.1 (1.1–2.2–7.5–19.4)	–6.431	<.001	123.114	<.001	0.030
*Model* 2							
Maximum diameter (mm)	14.0 (10.0–12.0–16.0–22.0)	22.3 (11.0–18.0–30.0–55.3)	–13.916	<.001	173.907	<.001	0.233
Orthogonal diameter (mm)	8.0 (5.0–7.0–10.0–14.0)	15.0 (7.3–11.0–20.0–38.8)	–15.273	<.001	180.943	<.001	0.240
Axial ratio	0.6 (0.4–0.5–0.7–0.9)	0.7 (0.4–0.6–0.8–0.9)	–6.816	<.001	51.559	<.001	0.082
CTavg (HU)	42.0 (19.7–33.2–49.0–60.4)	47.0 (26.3–40.0–51.0–63.0)	–4.412	<.001	12.804	<.001	0.022
SUVmax	5.2 (1.9–3.5–8.0–15.4)	7.9 (2.3–4.5–13.0–29.4)	–6.352	<.001	57.608	<.001	0.091
*Model* 3							
Maximum diameter (mm)	13.0 (7.0–10.0–15.0–22.0)	20.0 (9.6–14.0–26.9–56.5)	–9.683	<.001	14.479	<.001	0.146
Orthogonal diameter (mm)	9.0 (5.0–7.0–11.0–14.0)	14.0 (7.0–11.0–19.3–38.0)	–10.851	<.001	11.939	<0.001	0.150
Axial ratio	0.7 (0.5–0.6–0.8–0.9)	0.8 (0.5–0.6–0.8–0.9)	–2.501	.012	0.019	–	–
CTavg (HU)	45.0 (14.0–35.0–51.0–60.0)	46.0 (23.6–40.0–50.0–63.0)	–1.383	.167	0.058	.090	0.007
SUVmax	8.6 (3.4–6.1–13.0–20.0)	10.0 (2.8–6.3–17.5–32.1)	–1.878	.060	14.468	–	–
*Model* 4							
Maximum diameter (mm)	19.0 (13.3–16.0–22.8–26.4)	35.0 (19.9–27.7–52.0–120.2)	–6.341	<.001	27.620	<.001	0.289
Orthogonal diameter (mm)	10.0 (5.0–8.0–13.0–15.4)	22.5 (12.1–16.8–42.3–94.3)	–6.592	<.001	26.886	<.001	0.283
Axial ratio	0.5 (0.3–0.4–0.6–0.8)	0.7 (0.4–0.6–0.8–0.9)	–3.827	<.001	17.914	<.001	0.209
CTavg (HU)	41.0 (18.3–34.3–46.8–56.7)	45.1 (9.0–41.7–48.3–62.1)	–1.723	.085			
SUVmax	5.9 (2.4–3.7–11.2–17.7)	10.8 (3.4–6.1–19.8–31.0)	–3.007	.003	9.466	.003	0.122
*Model* 5							
Maximum diameter (mm)	12.0 (7.3–11.0–16.0–23.1)	23.0 (11.8–17.0–37.0–101.0)	–5.443	<.001	17.981	<.001	0.209
Orthogonal diameter (mm)	10.0 (4.3–8.0–11.0–15.4)	16.0 (8.9–13.0–23.0–92.4)	–5.464	<.001	13.961	<.001	0.170
Axial ratio	0.7 (0.5–0.7–0.8–1.0)	0.7 (0.4–0.6–0.8–1.0)	–0.318	.750			
CTavg (HU)	47.0 (21.5–35.3–51.8–61.8)	46.5 (8.9–40.0–51.3–63.8)	–0.142	.887			
SUVmax	11.7 (3.7–8.0–16.3–32.8)	13.3 (4.0–9.2–23.3–54.8)	–1.839	.066			

Model 1: the all affected LNs in each patient were considered as research object. Model 2: the 5 largest affected LNs in terms of maximum diameter in each patient were considered as research object. Model 3: the 5 largest affected LNs in terms of SUVmax in each patient were considered as research object. Model 4: the largest affected LNs in terms of maximum diameter in each patient were considered as research object. Model 5: the largest affected LNs in terms of SUVmax in each patient were considered as research object.

CTavg = average CT value, PET/CT = positron emission tomography/computed tomography, SUVmax = maximum standard uptake values.

* Pearson Chi-Square test.

† One-way analysis of variance test for connections between disease type and PET/CT parameters.

Based on results from exploratory analyses and according to the reliability, repeatability and reproducibility principle, age, fever, arthralgia, abnormal WBC, abnormal LDH, splenomegaly, perinodal infiltration, node necrosis, node conglomeration, and maximum diameter and SUVmax of affected LNs in model 2 were selected as differential diagnostic scheme items for distinguishing KFD from lymphoma. Based on ROC analyses, cutoff values of age, maximum diameter and SUVmax were 34.5 years (sensitivity = 78.9%, specificity = 78.1%, AUC = 0.824), 20.5 mm (sensitivity = 71.1%, specificity = 96.9%, AUC = 0.829) and 27.5 (sensitivity = 60.5%, specificity = 68.7%, AUC = 0.627), respectively. The final differential diagnostic scheme was based on the weighted sum of 11 items (Table 4). Age ≤ 34, with fever, arthralgia, abnormal WBC, abnormal LDH, without node necrosis and without node conglomeration, have a score of 2 each. Other items have score of 1 each. Individuals who meets the inclusion criteria, and does not have any condition listed as exclusion criteria, and who have a total score > 8 for the items above, meet the criteria for KFD. Sensitivity and specificity were high: 86.8% (95% CI: 71.9%, 95.5%) and 96.9% (95% CI: 83.7%, 99.5%), AUC = 0.975 (95% CI: 90.5%, 99.6%), respectively.

**Table 4:** A rough scheme for distinguishing Kikuchi-Fujimoto disease (KFD) from lymphoma. The classification of KFD applies to any individual who meets the inclusion criteria*, and does not have any condition listed as exclusion criteria†, and who have a total score > 8 when summing the score from the following items.

Item	Score
Age ≤ 34	2
Fever+	2
Arthralgia+	2
Abnormal WBC+	2
Abnormal LDH+	2
Splenomegaly+	1
Perinodal infiltration-	1
Node necrosis-	2
Node conglomeration-	2
Median maximum diameter‡ ≤ 20.5	1
Median SUVmax‡ ≤ 7.1	1

* Inclusion criteria: patients presenting enlarged LNs predominantly on the upper side of the diaphragm.

† Exclusion criteria: main affected region other than upper side of the diaphragm, with other LNs enlargement diseases, or lymphoma with solid organ involvement.

‡ Model: the 5 largest affected LNs in terms of maximum diameter in each patient were considered as research object.

## 4. Discussion

KFD and lymphoma are both known as LNs enlargement diseases. KFD is characterized by subacute necrotizing regional lymphadenopathy, predominantly in cervical region.^[1]^ KFD commonly affects young adults and is frequently associated with fever, and occasionally with other nonspecific systemic symptoms such as arthralgia, and upper respiratory symptoms.^[1]^ Laboratory findings and imaging studies are also nonspecific. These manifestations may include atypical lymphocytes, elevated serum lactate dehydrogenase (LDH), bilateral neck LNs enlargement.^[3]^ Lymphoma often presents as enlarged LNs in a single or multiple regions of nodes on one or both sides of diaphragm, and involves solid organs such as lung, bone or liver.^[14]^ However, when the patient with lymphoma only present as enlarged LNs predominantly in cervical region and without solid organ involvement, it can easily be confused with KFD.

In this study, both patients with KFD and patients with lymphoma were on the condition that presented as enlarged LNs predominantly on the upper side of the diaphragm. The diagnosis of KFD or lymphoma can be challenging. It is showed that there is a tendency that the patients with KFD are more likely to suffer fever than patients with lymphoma. Although lymphoma typically presents as fever, most patients with lymphoma in this study were without fever. It maybe that those patients presented as lymph node enlargement just on the upper side of the diaphragm and the affected LNs were not much enough leading to low tumor load, and thus there is not much enough cytokines, such as tumor necrosis factor-α which regulate the fever reaction through the hypothalamus.^[11]^ However, KFD is most commonly associated with fever (35–77% of cases) due to various infections or body’s autoimmune response to various stimuli including the bacterial and viral agents and dietary sources.^[15]^ There is also a possible role for interferon-gamma, interleukin-6 and apoptotic cell death.^[15]^

KFD usually affects young adults (younger than 40 years old) with female predominance, but it can occur in any age group.^[16–18]^ The median age of the patients with KFD in this study is of 27.0 (21.0–33.5) years old and is younger than patients with lymphoma as well. Furthermore, most of them are female. Most patients with KFD usually have normal laboratory findings. In some cases, there is leukopenia (especially granulocytopenia; 20–58% of cases), leukocytosis (2–5% of cases) and elevated serum LDH.^[1]^ In this study, about 46.9% of KFD patients were with leukopenia and 25.0% were with leukocytosis. 84.4% of them were with elevated serum LDH. It was confirmed that the incidence of abnormal WBC or LDH of patient with KFD was higher than that of patient with lymphoma. It implied that the patient with abnormal WBC or LDH should be more likely to be KFD on the condition of this study.

Patients with KFD most commonly present with cervical lymphadenopathy, frequently with concomitant involvement of axillary and/or supraclavicular LNs. In this study, up to 75.0% of affected LNs were in *Head and Neck*. In addition, level II was the most frequent site, followed by level V, which was in accordance with previous research.^[4]^ Lymphadenopathy almost always presents tender and painful LNs which can be enlarged in size to 5–35 mm, typically < 40 mm.^[1,2]^ But it may reach 50 to 60 mm and rarely larger than 60 mm. In this study, the maximum diameter of affected LNs in patients with KFD ranged from 2 to 27 mm. These results are in accordance with previous reports. In addition, all of these results are smaller than that of lymphoma in each model. Furthermore, the maximum diameter and orthogonal diameter of affected LNs were intensely associated with the disease type in each model. There are about 12.5% KFD patients and 13.2% lymphoma patients with LNs pain. 28.1% KFD patients are with arthralgia and no lymphoma patient with arthralgia. It is reported that hepatomegaly and splenomegaly rarely occur (<5% of cases) in patients with KFD.^[1]^ However, more than 34.4% and 81.3% KFD patients are with hepatomegaly and splenomegaly, respectively. Further analysis in large sample studies is necessary. Although it was reported that most affected LNs were with perinodal infiltration (90%), lymph node necrosis (55%) and lymph node conglomeration (75%) for KFD,^[19]^ only a few KFD patients were with those characteristics in this study. The different results may be credited to that the severity of nodal perinodal infiltration, necrosis and node conglomeration were analyzed using a contrast enhancement CT pattern in the previous research, while those characteristics were analyzed using a plain low-dose CT pattern (with lower spatial resolution and contrast resolution) in this study. In addition, despite the CTavg in model 1 and model 2 were lower in patients with KFD than that in patients with lymphoma, the difference of those results between the 2 group patients was slight. Furthermore, no significant differences were observed between the 2 group patients in CTavg of affected LN in model 3, 4, and 5. These findings may be result from most affected LNs without necrosis both in KFD (96.9%) and lymphoma (63.2%) in this study.

^18^F-FDG PET/CT, a noninvasive technique, is useful for differentiating between benign and malignant tumors, where the SUVmax of ^18^F-FDG PET images correlate with malignant tumor aggressiveness. However, some benign tumors as well as inflammations (tuberculosis, sarcoidosis) can have quite high values of SUVmax. It suggested that the affected LNs of patients with KFD has a tendency of high ^18^F-FDG uptake even though relatively small.^[9]^ In this study, the value of SUVmax was up to 50.0 in patients with KFD. FDG avidity in affected LNs may be due to increased glycolysis and glucose transporter activity of proliferated histiocytes and necrotizing lymphocytes and numerous histiocytes surrounding the necrotic foci.^[9]^ The SUVmax of affected LNs in model 1, 2, and model 4 were both lower in patients with KFD than that in patients with lymphoma. Because of maximum diameter, orthogonal diameter, and SUVmax were strong different between the 2 group patients, it implied that model 2 was more suitable for differentiating between KFD and lymphoma.

According to the present study, we found that the clinical features (age, fever, arthralgia, abnormal WBC, abnormal LDH) and PET/CT features (splenomegaly, perinodal infiltration, node necrosis, node conglomeration, and maximum diameter and SUVmax of affected LNs in model 2) were helpful in making the differential diagnosis between KFD and lymphoma. Together with those parameters may be useful for suggesting the possibility of KFD. A rough differential diagnostic scheme for distinguishing KFD from lymphoma was developed: individuals who meets the inclusion criteria, and does not have any condition listed as exclusion criteria, and who have a total score > 8 for the items above.

A limitation of our study was the small size of the study population due to the low prevalence of KFD and the high cost of ^18^F-FDG PET/CT. Nevertheless, this limitation is unlikely to affect our conclusion as previous case reports were consistent with our results. In spite of this limitation, our study has pioneered, to the best of our knowledge, evaluating the potential value of clinical and PET/CT features for differentiating KFD and lymphoma on the condition that patients presenting enlarged LNs predominantly on the upper side of the diaphragm.

In conclusion, the awareness of clinical characteristics and spectrum of ^18^F-FDG PET/CT findings can be beneficial to differentiate KFD from lymphoma, particularly in patients with lymphadenopathy predominantly on the upper side of the diaphragm.

## Acknowledgments

We gratefully thank the technicians in our department for their help.

## Author contributions

**Conceptualization:** Mu-Hua Cheng, Liang-Jun Xie.

**Data curation:** Liang-Jun Xie.

**Formal analysis:** Liang-Jun Xie.

**Investigation:** Liang-Jun Xie.

**Methodology:** Mu-Hua Cheng, Liang-Jun Xie.

**Writing – original draft:** Liang-Jun Xie.

**Writing – review & editing:** Mu-Hua Cheng, Liang-Jun Xie.
